# PROTOCOL: Exercise interventions to improve back shape/posture, balance, falls and fear of falling in older adults with hyperkyphosis: A systematic review

**DOI:** 10.1002/cl2.1101

**Published:** 2020-07-12

**Authors:** Roongtip Duangkaew, Josette Bettany‐Saltikov, Paul van Schaik, Gokulakannan Kandasamy, Julie Hogg

**Affiliations:** ^1^ School of Health and Life Sciences Teesside University Middlesbrough UK; ^2^ Department of Physical Therapy, Faculty of Allied Health Sciences Thammasat University Pathumthani Thailand; ^3^ School of Social Sciences, Humanities and Law Teesside University Middlesbrough UK

## Abstract

**Aim:**

The aim of this systematic review is to evaluate and synthesize published and unpublished literature on the effectiveness of a diverse range of exercise programs on back shape/posture, balance, falling and fear of falling in older people with hyperkyphosis.

**Objectives:**

The objective of this systematic review is to determine the effects of difference exercise programs on back shape/posture, balance, falling and fear of falling in older adults with hyperkyphosis.

## 1 BACKGROUND

### 1.1 The problem, condition or issue

Spinal posture changes with age and typically results in exaggerated curves in the sagittal plane, the frontal plane or in both. Hyperkyphosis, also known as the "dowager's hump", is the most common spinal deformity in older adults. Hyperkyphosis is defined as an excessive forward curvature of the thoracic spine in the sagittal plane (from the side) when a kyphosis angle is equal or greater than 40° using the Cobb method (measured on a standing lateral spinal x‐ray generally measured from the 4^th^ to the 12^th^ thoracic vertebrae (Fon, Pitt, & Thies, 1980; Roghani, Zavieh, Manshadi, King, & Katzman, 2017; Voutsinas & MacEwen, 1986). The normal range of kyphosis in young adults ranges from 20° to 40° of the thoracic curvature (Fon et al., 1980). Thoracic kyphosis tends to progress with age (Milne & Lauder, 1974) and affect both sexes (Katzman et al., 2016). The kyphosis angle increases more rapidly in women than men after 40 years of age (Ensrud et al., 1997), with the mean kyphosis angle ranging between 44° and 48° (Ensrud et al., 1997; Schneider, von Mühlen, Barrett‐Connor, & Sartoris, 2004). In terms of biomechanical dysfunction, an increased thoracic kyphosis alters the vertebral column alignment in the sagittal curve of the spine, which leads to an increase in mechanical loading, flexion moments, compression and shear force on the spine (Balzini et al., 2003; Mika, Unnithan, & Mika, 2005; Pearsaii & Reid, 1992). In addition, changes in spinal shape and contour limit the movement and mobility of the rib cage (Horie et al., 2009), resulting in poor balance (Lynn, Sinaki, & Westerlind, 1997), functional limitations (Ryan & Fried, 1997) as well as increased mortality (Kado, Huang, Karlamangla, Barrett‐Connor, & Greendale, 2004), all of which significantly affect the health and quality of life of older adults (Horie et al., 2009).

#### 1.1.1 Aetiology of hyperkyphosis

To date, the aetiology or causation of hyperkyphosis in older adults remains unclear (Kado, Prenovost, & Crandall, 2007; Katzman, Wanek, Shepherd, & Sellmeyer, 2010; Roghani et al., 2017). Many theories on the causes of hyperkyphosis have been proposed, such as multiple musculoskeletal, neuromuscular and different sensory impairments (Katzman et al., 2010). The different range of aetiologies associated with hyperkyphosis includes vertebral fractures and low bone mineral density, degenerative disc disease, back extensor muscle weakness, calcification and ossification of intervertebral ligaments as well as decreased mobility of the spine, together with proprioceptive and sensory deficits and genetics.

Bouxsein et al. (2006) demonstrated that the stress loading on the vertebral spine during activities of daily living can gradually affect vertebral wedging and increase compression fractures. In addition, the severity of vertebral wedging increases as bone mineral density decreases (Goh, Price, Leedman, & Singer, 1999; Milne & Lauder, 1976).

Degenerative disc disease is another causative factor that may affect the development of hyperkyphosis. Schneider et al. (2004) reported that degenerative disc disease is a common radiographic finding associated with hyperkyphosis in older adults. Moreover, Manns, Haddaway, McCall, Pullicino, and Davie (1996) documented a significant correlation between anterior disc height and kyphosis angle; as the anterior disc height decreased, the angle of kyphosis increased.

Dastmanesh, Eskandari, and Shafiee (2013), Granito, Aveiro, Renno, Oishi, and Driusso (2012), Hirano et al. (2013) and Mika et al. (2005) have also suggested that there is an inverse correlation between the kyphosis angle and back extensor muscle weakness in adolescence and older adults.

Furthermore, there are two theories that have been proposed for describing back extensor muscle weakness. The first theory relates to the reduction of the capability of back muscles to generate an extension moment and control shear force which can result in an increased thoracic kyphosis (Greig, Bennell, Briggs, & Hodges, 2008). The second theory relates to an increase in the anterior angle of the thoracic spine that leads to an increase in both a compression force as well as a shear force on the thoracic spine (Pearsaii & Reid, 1992). Further, decreased spinal mobility together with the degeneration of the intervertebral ligaments occurs with ageing and disturbs the ability of the spine to control normal postural alignment (Kado et al., 2007; Katzman et al., 2010; Roghani et al., 2017).

Furthermore, several systems within the body, including the proprioceptive, the visual and the vestibular systems contribute towards maintaining an upright position. As ageing results in a decline in all these systems, this consequently leads to the loss of upright postural control (Ferrucci et al., 2004; Katzman et al., 2010; Roghani et al., 2017).

Hyperkyphosis can also be hereditary. Kado et al. (2007) and Roghani et al. (2017) reported that elderly people who had a family history of hyperkyphosis were statistically more likely to have a greater kyphosis than those without such a family history.

#### 1.1.2 Prevalence of hyperkyphosis

The estimated prevalence of hyperkyphosis has been reported to be 20–40% of the older adult population aged over 60 years (Kado et al., 2004; Katzman, Sellmeyer, Stewart, Wanek, & Hamel, 2007; Ryan & Fried, 1997; Takahashi et al., 2005). This deformity affects both sexes, particularly women aged over 55 years (Huang, Barrett‐Connor, Greendale, & Kado, 2006; Katzman et al., 2010), regardless of vertebral fractures, with the incidence increasing to 6–11% for every 10‐year increase in age (Huang et al., 2006). For instance, Kobayashi, Atsuta, Matsuno, and Takeda (2004) in a longitudinal study of 100 healthy males and females aged 50 years or older, the authors reported a mean thoracic angle increase of 3° per decade. Kado et al. (2007) study on men and women reported a mean thoracic kyphosis angle of 26° in persons in their 20s and a mean thoracic angle of 53° in those 60–74 years of age and 66° in those older than 75 years of age.

#### 1.1.3 Consequences of hyperkyphosis

Hyperkyphosis has been associated with impaired pulmonary function, functional limitations, falls, fractures, a decreased quality of life as well as increased mortality (Kado et al.,^2004^; Katzman et al., 2010). The ageing process combined with thoracic kyphosis in older adults may restrict thoracic inspiration capacity and lead to impaired pulmonary function (Kado, 2009). Furthermore, Kado (2009) demonstrated that women with hyperkyphosis and vertebral fractures had a greater mortality rate than women affected by either hyperkyphosis or vertebral fractures alone.

In addition, Takahashi et al. (2005) investigated the association between spinal deformities in the sagittal plane and functional impairment of daily living in community‐dwelling subjects. The authors reported that older adults with hyperkyphosis reported having significant limitations during daily activities and less satisfaction with life in general. Accentuated thoracic kyphosis is also known to cause displacement of the centre of gravity that can lead to impaired balance and increased risk of falling (Lynn et al., 1997). Several studies have reported that hyperkyphosis is associated with an increased premature death (Kado, 2009; Milne & Williamson, 1983). Hyperkyphosis is also known to be an indicator of the negative impact of health outcomes.

### 1.2 The intervention

The management of age‐related hyperkyphosis includes exercises, spinal orthosis and surgery (Bansal, Katzman, & Giangregorio, 2014). Exercise training is a common approach that aims to increase spinal muscle strength and stretching exercises in an attempt to the realignment posture and reduce hyperkyphosis (Bettany‐Saltikov, Turnbull, Ng, & Webb, 2017). Exercise‐based interventions may include spinal muscle strengthening, core stabilisation exercises as well as stretching exercises. Spinal muscle strengthening refers to weight‐bearing and resistance exercises of the spine; an increase in spinal muscle strength, which helps to maintain the spine in the upright position (Ball, Cagle, Johnson, Lucasey, & Lukert, 2009). Core stabilisation exercises can be described as the ability of the neuromuscular system to control the position and motion of the trunk over the pelvis and legs in order to allow the optimum production, transfer and control of forces as well as motion to provide stabilisation by controlling spinal movement (Kibler, Press, & Sciascia, 2006).

Stretching exercises are a type of a therapeutic exercise designed to increase the extensibility of soft tissues, thereby improving spinal flexibility by elongating (lengthening) structures that have adaptively shortened and become hypomobile over time (Kisner, Colby, & Borstad, 2018).

There are differnt types of thoracic hyperkyphosis curves. Hyperkyphosis‐specific exercises for curve types of hyperkyphosis are needed for physiotherapists to treat their patients effectively. The exercises which include overcorrection of the spine in the sagittal plane, stretch shortened muscles and strengthen longer muscles that are used to address the different curve types of hyperkyphosis (Bettany‐Saltikov, Turnbull, Ng & Webb, 2017). Weiss & Turnbull, 2010 demonstrated that hyperkyphosis‐specific exercises are effective for various types of hyperkyphosis in an outpatient setting.

The purpose of the exercise is three‐fold. Firstly to improve the thoracic hyperkyphosis, secondly, to delay its progression and thirdly to alleviate the resultant accompanying complications due to hyperkyphosis in older people (Ailon, Shaffrey, Lenke, Harrop, & Smith, 2015).

## 2 HOW THE INTERVENTION MIGHT WORK

The ageing process is associated with numerous alterations in posture that include changes to hip flexion and external rotation, needing a wide base of support, a forward head posture and increased thoracic kyphosis (Woodhull‐McNeal, 1992). Hyperkyphosis or excessive curvature in the thoracic spine in the sagittal plane leads to shifts in the centre of gravity and brings the body closer to the edge of the base of support, resulting in a decreased ability to balance and there by increasing the risk and number of falls (Danis, Krebs, Gill‐Body, & Sahrmann, 1998). To date, postural training, spinal extensor muscle strength training as well as spinal flexibility exercises have been a significant focus of exercise therapy in the management of patients with hyperkyphosis (Bansal et al., 2014).

The goals of corrective exercises include the following: to decrease spinal pain, to reduce the hyperkyphotic posture, to increase spinal mobility as well as to improve the person's quality of life. Exercise protocols are generally aimed at correcting posture, increasing back extensor muscle strength as well as increasing the core stability of the trunk (Bansal et al., 2014; Hsu, Chen, Tsauo, & Yang, 2014; Kado, 2009).

Corrective postural exercises are based on Kendall's theory that suggests that back extensor exercises are able to reduce the angle of kyphosis with the strong back muscles counteracting the anteriorly directed gravitational pull on the thoracic spine (Ball et al., 2009; Kendall, McCreary, & Provance, 2005). They consist of stretching exercises to the anterior aspect of the trunk combined with strengthening exercises to the posterior aspect of the trunk (Seidi, Rajabi, Ebrahimi, Alizadeh, & Minoonejad, 2014). Core stability exercises, such as yoga, Pilates and Tai Chi, are considered to consist of a "muscular box" with the abdominals in the front, paraspinals and gluteals in the back, the diaphragm as the roof and the pelvic floor and the hip girdle musculature as the bottom (Akuthota & Nadler, 2004; Akuthota, Ferreiro, Moore, & Fredericson, 2008). This "muscular box" works as a stabiliser for the spine and pelvis which consequently form a kinetic chain during functional movement. These support the upright position and assist balance while walking. Consequently, these muscles function to reduce the risk of falls and improve the persons quality of life (Sinaki et al., 2002). However, most currently available exercises (Ball, Cagle, Johnson, Lucasey, & Lukert, 2009; Greendale, McDivit, Carpenter, Seeger, and Huang, 2002) address only one or two dimension planes.

According to Negrini et al. (2005), scoliosis‐specific exercises were suggested as the first step in the treatment of adolescent patients with hyperkyphosis and scoliosis. Numerous approaches to scoliosis‐specific exercises or "Schools" are available. These scoliosis‐specific exercises comprise of the SEAS approach (Scientific Exercise Approach to Scoliosis); the Schroth method (original Schroth); Schroth Best Practice; the BSPTS (Barcelona Scoliosis Physical Therapy School); the Dobomed method; FITS (Functional Individual Therapy of Scoliosis); the Lyon method as well as Min Mehta's 'Side‐shift' exercises (Bettany‐Saltikov et al., 2017). The principles of these exercises include improving the patient's awareness of their deformity to promote self‐correction; repeated 3D spinal correction exercises, which involves trunk elongation, pelvic alignment, side‐shift of thorax, shoulder corrections and derotation with breathing in lying, sitting and standing positions, spinal mobilisation; active 3D stabilisation; corrective breathing; repetition to correct body schema as well as the integration of postural corrections within activities of daily living (Bettany‐Saltikov, Parent, Romano, Villagrasa, & Negrini, 2014). Berdishevsky (2016) conducted a case study to investigate the effects of an intensive physiotherapy programme (the Barcelona Scoliosis Physical Therapy School, which is a derivative of the Schroth approach) together with a SpinoMed brace (symmetrical brace) on the kyphosis Cobb angle, pain, quality of life and spinal muscles strength on an older adult woman with Scheuermann's kyphosis aged 76 years old. The researcher found that an intensive physiotherapy programme based on the Barcelona Scoliosis Physical Therapy School and SpinoMed brace was effective for the treatment of an older adult with Scheuermann's kyphosis. However, the generalisation of the results of this study was limited due to the involvement of only a single participant. To the best of the authors' knowledge, there are no studies that have examined the effects of the Schroth method on back shape/posture, balance, falls, and fear of falling in older adults with age‐related hyperkyphosis.

## 3 WHY IT IS IMPORTANT TO DO THIS REVIEW

A scoping search identified four narrative reviews that have been reported on exercise‐based interventions as part of the treatment or management of age‐related hyperkyphosis. Ailon et al. (2015), reviewed the progression of spinal kyphosis in the ageing population and suggested that exercise interventions can improve thoracic hyperkyphosis, physical functioning and back extensor muscle strength. Conversely, several studies have not found any difference in the kyphosis angle following exercise (Greendale, McDivit, Carpenter, Seeger, & Huang, 2002; Itoi and Sinaki, 1994). Thus, the efficacy of exercise programmes for hyperkyphosis demonstrates conflicting results.

Furthermore, Kado (2009) and Kado et al. (2007) reviewed the rehabilitation of having a hyperkyphotic posture in the elderly. The researchers demonstrated that there are numerous exercise interventions such as back strengthening exercises, aerobic exercises, spinal extension exercises, yoga, pilates as well as postural alignment training for improving hyperkyphosis. All these types of exercises focus on back strength as well as the outcomes of correcting the kyphosis. In addition, Katzman et al. (2010) revealed the indications and contraindications of performing exercises for hyperkyphosis. The investigators revealed that flexion exercises tended to increase the risk for vertebral fractures in participants with underlying osteoporosis and vertebral fractures. Hence, flexion exercises or flexion stresses on the spine during exercise or activities daily living need to be avoided and trunk extension exercises are recommended instead.

Further, a systematic review by Bansal et al. (2014) examined whether exercise was able to reduce the angle of thoracic kyphosis in older adults aged ≥45. The authors found that the existing evidence concerning the effects of exercise on hyperkyphosis in elderly people were scarce and mostly of low quality. Furthermore, a recent systematic review by González‐Gálvez, Gea‐García, and Marcos‐Pardo (2019) assessed the effect of the exercise interventions on the subjects thoracic kyphosis and lumbar lordosis angle. The authors revealed that exercise programmes may have a positive effect on the thoracic kyphosis angle, but we are not wheather they has any effect on the lumbar lordotic angle. In summary, previous narrative and systematic reviews (Ailon et al., 2015; Bansal et al., 2014; González‐Gálvez et al., 2019; Kado et al., 2007; Kado, 2009; Katzman et al., 2010) did not specially focus on balance, falls, and fear of falling issues. Therefore, this review is urgently needed to improve the management of older people with hyperkyphosis; in an attempt at improving thoracic kyphosis, balance, falling and fear of falling in the elderly population.

In addition, In 2013 the National Institute for Health and Care Excellence (NICE) published clinical guidelines regarding the assessment of risk as well as the prevention of falls in older people for health care and other professionals. According to the guideline recommendations, a multifactorial intervention program for older adults with recurrent falls or assessed as being at increased risk of falling included muscles strengthening and balance training, home hazards assessment and intervention, vision assessment and referral as well as medication review with modification/withdrawal. However, the multifactorial intervention program does not currently include exercises programs for preventing falls in older adults with hyperkyphosis.

This current review aims to gather existing research evidence on the effectiveness of exercise interventions for treating older adults with hyperkyphosis. This review may enable clinicians and researchers as well as policy makers involved in the management of hyperkyphosis in older adults that exercise programs could potentially be included within this guideline/or policy for the prevention falls.

Therefore, considering all the above we believe that this reivew is urgently needed to improve both the current health policies as well as the treatment interventions for the management of older people with hyperkyphosis, specifically in attempting to improve the degree of thoracic kyphosis, balance, falling, and fear of fallingin the elderly population.

## 4 OBJECTIVES

### 4.1 Aims

The aim of this systematic review is to evaluate and synthesise both published and unpublished literature on the effectiveness of a diverse range of exercise programmes on back shape/posture, balance, falling and fear of falling in older people with hyperkyphosis.

### 4.2 Objectives

This objective of this systematic review is to determine the effects of different exercise programmes on back shape/posture, balance, falling and fear of falling in older adults with hyperkyphosis.

## 5 METHODS

### 5.1 Criteria for considering studies for this review

#### 5.1.1 Types of studies

Types of intervention studies that will be reviewed include the following:

- Randomised controlled trials.
- Quasi‐experimental designs.
- Pre‐post intervention studies.

Types of intervention studies that will be excluded in the review:

- Cross‐sectional studies.
- Case‐control studies.
- Cohort studies.
- Qualitative studies.

#### 5.1.2 Types of participants

*Studies that will be included in the review:*

- Participants will be older adults (female or male).
- Aged 50 years or older.
- Participants who have been diagnosed or identified as having a thoracic hyperkyphosis (hyperkyphosis is defined as an excessive anterior curvature of thoracic spine,a kyphosis angle greater than 40° when measured with the Cobb method (Fon et al., 1980; Voutsinas & MacEwen, 1986).

*Studies will be excluded:*

- Participants are adults who are aged 49 years and under or children as well as adolescents.
- Participants who have other spinal deformities such as scoliosis, kyphoscoliosis, Scheuermann's kyphosis and congenital kyphosis.

#### 5.1.3 Types of interventions

The intervention in the study must meet the following criteria:

- Exercise intervention.
- Therapeutic exercise.

Studies will be excluded if they do not include a type of exercise intervention. For example, these are not exercise intervention: spinal orthoses, postural taping and spinal surgery.

#### 5.1.4 Types of outcome measures

Balance, falls, fear of falling and back shape/posture (such as thoracic kyphosis angle, lumbar lordosis angle, pelvic tilt) will be considered to be the outcomes in this review.

##### Primary outcomes

1. Back shape/posture will be measured using several methods as follows: the lateral spine radiographs, a computerised measurement device for surface curvature (SpinalMouse®), Debrunner's kyphometer, manual inclinometer, digital inclinometer, goniometer, electrogoniometry, photogrammetry, microscribe digitiser, computer apps, Integrated Shape Imaging System, six‐camera motion analysis system as well as flexicurve method.

Back shape/posture will be assessed by:

*Method*

- Cobb angle in degrees (absolute values).
- SpinalMouse® method in degrees (absolute values).
- Flexicurve method in degrees (absolute values).

Further, back shape/posture also includes variable parameters as follows: Sagittal plane

- Cervical lordosis in degree (absolute values).
- Thoracic kyphosis in degrees (absolute values).
- Lumbar lordosis in degrees (absolute values).

Studies will be excluded if they do not measure back shape, posture in degree. For example, the blocks method, the occiput‐wall distance method, and so forth.

2. The measurement of balance need to include the measurement of postural sway, together with the values limits for the limits of stability.

- Postural sway is defined as the movement of the centre of mass in quiet standing and can be measured as anteroposterior and mediolateral displacement. This will be obtained with the use of a computerised force platform.
- The limits of stability are defined as the maximum range in which the centre of gravity can move safely without either moving their feet and without falling (Alexander, 1994).

3. Fear of falling is identified as older people lose their confidence when participating in activities of daily living. Older adults are concerned about the risk of falling that both limits their daily activities as well as functioning (Tinetti & Powell, 1993).

4. Falls are defined as a 'history of falls' with at least one fall in the past year. A fall is defined as an unexpected event in which the participant come to rest on the ground, floor or lower level (Lamb, Jørstad‐Stein, Hauer, Becker, & Prevention of Falls Network Europe and Outcomes Consensus Group, 2005). History of falls or falls (number of falls/year) is measured by asking the participants the number of falls that they have in the past 12 months or 6 months.

The details required for theoutcome measures are provided in Table 1.

**Table 1:** Lists of outcome measures

Outcome measures	Definition
Postural sway	Timed track length (millimetre/second; mm/s) is defined as the total distance travelled by the centre of pressure divided by the time indicating sway speed
	Enveloped area (square millimetre; mm^2^) is defined as the total area enveloped by the tract of the centre of pressure indicating the spatial spread of swaying
	Rectangular area (square millimetre; mm^2^) is defined as the rectangular area covered by the track of the centre of pressure, calculated as the product of maximal anteroposterior and right‐left distances of the track, expressing the two dimensional‐extent of swaying
	Deviation of lateral sway origin (millimetre) is defined as the deviation of right‐left sway origin indicating lateral deviation of bal
	Timed track length in the lateral direction (millimetre/second; mm/s) is defined as the total right‐left distance travelled by the centre of pressure divided by time indicating lateral sway speed
	Deviation of anteroposterior sway origin measured in millimetres is defined as the anteroposterior deviation of balance
	Timed track length in the anteroposterior direction (millimetre/second; mm/s) is defined as the total anteroposterior travelled by the centre of pressure divided by time indicating anteroposterior sway speed
	The displacement of the centre of pressure in the anteroposterior direction and the mediolateral direction (centimetre)
	Anterior‐posterior sway envelope (equilibrium score) is measured using computerised posturography
	The centre of pressure sway velocity (degrees per second)
	The position of the centre of pressure (degrees and percentages of the limit of stability)
	The 100% limits of stability (ability to shift their centre of pressure without losing balance; percentages of the limits of stability)
	Studies will be excluded if they measure functional balance:
	Functional balance measurements (e.g., Berg Balance Scale, Timed up and go, single‐leg stance time etc.) will be excluded. It is characterised by the tests which objectively measure a person performing a balance or walking task; however, these tests do not quantify postural sway
The limits of stability	The maximum displacement of the centre of pressure excursion in the forward direction (anterior), backward direction (posterior), right side direction (lateral) as well as left side direction (medial). The unit of the limits of stability will be measured in millimetre (mm) or centimetre (cm)
	To control for foot length, the functional stability limits are calculated and computed as the peak anteroposterior limits of stability as a per cent of foot length (%)
	Reaction time is defined as the time between the stimulus to move and actual response movement (second)
	Velocity of movement in the forward direction (anterior), backward direction (posterior), right‐side direction (lateral) as well as left side direction (medial). Unit of the limits of stability is measured in centimetre/second
Falls	History of falls or falls (number of falls/year) is measured by asking the participants the number of falls, that they had in the past 12 months.
	History of falls or falls (number of falls/year) is measured by asking the participants the number of falls, that they had in the past 6 months
Fear of falling	Fall efficacy scale (Total score = 100) is a 10‐item questionnaire. The scoring is a 10‐point scale for each item; 0 score means low fall‐related self‐efficacy, 100 means high fall‐related self‐efficacy (Gillespie & Friedman, 2007)
	Modified Fall efficacy scale (Total score = 140) is a 14‐item question that is developed from Fall efficacy scale and the scoring is also 10‐point visual analogue scale for each item; 0 = no confident/not sure at all, 5 = fair confident/fairly sure and 10 = complete confidence/complete sure. Higher scores indicate more confidence/less fear of falling, lower scores indicate less confidence/more fear of falling (Hill, Schwarz, Kalogeropoulos and Gibson, 1996)
	Fall efficacy scale‐International (Total score = 64) is a 16‐item questionnaire that will be rated on 4‐point Likert scale with the following possible answers: not at all = 1 point, somewhat = 2 points, fairly = 3 points and very concerned = 4 points. The high scores indicate a high fear of falling. The level fear of falling could be divided into three categories: low (FES‐I score 16–19), moderate (FES‐I score 20–27), high (FES‐I score 28 ‐ 64; Greenberg, 2011)
	Fear of falling will be measured using a visual analogue scale (VAS) on a vertical line of 10 cm and connecting the two statements no fear of falling (below) and very afraid of falling (above; Hill et al., 1996)
	Fear of falling will be measured using the following question: At the present time, are you very fearful, somewhat fearful or not fearful that you may fall? (Arfken, Lach, Birge, & Miller, 1994)

##### Secondary outcomes

###### Duration of follow‐up

There will be no restrictions regarding the duration of follow up outcomes in the review.

###### Types of settings

Included studies need to be conducted in any physiotherapy laboratory setting and home‐based setting.

### 5.2 Search methods for identification of studies

#### 5.2.1 Electronic searches

1. Electronic database searches

Relevant studies will be identified through the main health databases. The searches will include studies published from inception to the present. No language limitation will be applied to the searches.

The following databases will be searched:

- MEDLINE (1950–present)
- AMED (1985–present)
- CINAHL (1982–present)
- PEDro (1929–present)
- EMBASE (1974–present)
- Sport Discus (1892–present)
- Web of Science (1982–present)
- SCOPUS (1995–present)
- Cochrane Library (CENTRAL)

Google Scholar, a web search using search engines will be included in this review.

##### Search terms

Appendix 1 illustrates the search strategy for the MEDLINE database searched on the EBSCOhost platform.

We will modify the search terms and strategies for the different databases.

#### 5.2.2 Searching other resources

The following strategies will also be used:

1. Screening the reference lists of all relevant papers.
2. Searching of the main electronic sources of ongoing trials.
3. Searching the grey literature including dissertations theses and conference proceedings.
4. Contact leading authors for information on unpublished or ongoing trials.

### 5.3 Data collection and analysis

#### 5.3.1 Description of methods used in primary research

Anticipated methods that primary studies are likely to employ include randomised controlled trials, quasi‐experimental designs or pretest/posttest designs. We expect that the treatment conditions will be compared to either a no‐treatment control group or a comparison group receiving a different intervention.

#### 5.3.2 Criteria for determination of independent findings

We anticipate two issues relating to the determination of independent findings that will need to be addressed in this review. First, documents may report on multiple studies and/or multiple outcomes. Our protocol for this situation will be to allow documents to contribute multiple effect sizes but only contribute one effect size for each outcome. If a document provides multiple effect sizes for one outcome, the mean effect size for that outcome will be calculated using a Comprehensive Meta‐Analysis software package. The second issue of independence is where multiple documents report data from the same evaluation. We will treat dependent studies as a single study and use all sources to calculate the effect size for each outcome.

#### 5.3.3 Selection of studies

A data selection form will first be developed on the basis of the inclusion criteria and will then be piloted and tested before use by two review authors. Two review authors (RD and JB‐S) will independently screen the titles and abstracts. Potentially relevant studies and descriptors of identified studies for possible inclusion. From the full text, two review authors (RD and JB‐S) will assess potentially eligible trials for inclusion. If there will disagreement between review authors, a third reviewer author (PvS or GK) will be contacted to solve a disagreement.

#### 5.3.4 Data extraction and management

Two reviewers (RD and JBS) will independently extract data from relevant studies using a standard data extraction form, which will be developed by the authors and piloted before use. In the case of differences in the extracted data by the two reviewers, we will discuss these to reach a consensus, and if unresolved, these will be discussed with a third author. In the case of missing data, we will contact the original study author for clarification.

Data on the following will be extracted from included studies:

##### Participants

- Age
- Gender
- Comorbid conditions such as osteoporosis and vertebral compression fractures.

##### Intervention

- Type of exercise interventions and documented details including.
- Duration/intensity/frequency of the intervention.

##### Comparison intervention

- No intervention
- Other interventions

##### Outcomes

- Back shape/posture
- Balance measurement
- Falls
- Fear of falling

#### 5.3.5 Assessment of risk of bias in included studies

The risk of bias assessment will be performed by two independent authors (RD and JB‐S). This evaluation will be done using the Cochrane Collaboration's Risk of Bias tool (Higgins & Green, 2011), together with items from Law et al. (1998) (the McMaster University Critical Review Form for Quantitative Studies). The following criteria will be used to evaluate the risk of bias: random sequence generation; allocation concealment; blinding of participants and personnel; blinding of outcomes assessors; incomplete outcome data; and selective outcome reporting. We will review each study for the presence and absence of each criterion, and the score these as either high risk of bias, low risk of bias or unclear risk of bias (uncertain risk of bias). The McMaster University Critical Review Form for Quantitative Studies tool consists of eight domain questions (15 items) which assess the study purpose, the literature, the study design, the sample, the outcomes, the intervention, results, conclusion and the implications of the study. The answer to each question will be assessed as either yes, no, not applicable or not addressed. Each item, with a score of 2 will indicate the fulfilment of the question answered in full, a score of 1 will indicate a lack of the detail and a score of 0 will indicate nonfulfilment of the detail. This critical appraisal tool will be chosen as it can be used for a variety of study designs. Any disagreements arising between the reviewers being resolved by consensus and discussion within the review team (PvS and GK).

#### 5.3.6 Measures of treatment effect

##### Dichotomous outcomes

For dichotomous outcomes, we will calculate the odds ratios with a 95% confidence interval (CI) to summarise the results of each study. For the purpose of meta‐analysis, we will tranform the values to Hedge's g.

##### Continuous outcomes

The calculation of effect sizes will take into account the variety of measures used to assess intervention effect by calculating and analyzing all outcomes using the standardized mean difference (SMD) converted to Hedges' *gto account for small sample sizes. All effect sizes will be calculated using the 95% confidence interval (CI). Studies may present their results using statistics such as standard errors, confidence intervals, T‐values, or p‐values, effect sizes will be computed using appropriate conversion conventions provided by the Comprehensive Meta‐analysis (CMA) software package*.

#### 5.3.7 Unit of analysis issues

In this systematic review, it is anticipated that included studies that may have three or more interventions are evaluated in a single study. We will include each pair‐wise comparison separately. In cases of multiple time points, we will analyze each outcome at each time point separately. Comparable studies taking measures at a similar time point will be analyzed together, grouped as follows: short‐term (less than 6 months after intervention), medium‐term (6 months to less than 12 months after intervention) and long‐term (12 months or more after intervention).

#### 5.3.8 Dealing with missing data

Missing data or study dropouts for each of the included studies will be documented. We will contact the study authors requesting the required data. When data are insufficient to be entered into the meta‐analysis (even after contacting the authors), we will report the results qualitatively in the "table of characteristics of Included studies" and in the "summary of findings tables".

#### 5.3.9 Assessment of heterogeneity

Heterogeneity will be assessed by comparing factors such as participant demographics, type of intervention and outcome measures. Statistical heterogeneity will be assessed visually and by examined using the *I* ^2^ statistic, which describes the approximate proportion of variation that is due to heterogeneity rather than sampling error. This will be supplemented by the *χ* ^2^ test, where a *p* < .05 indicates significant statistical heterogeneity of intervention effects. Moreover, we will estimate and present *T* ^2^, along with its CIs, as an estimate of the magnitude of variation between studies. This will provide an estimate of the amount of between study variation.

#### 5.3.10 Assessment of reporting biases

We will assess reporting biases to determine whether publication bias is present and we will construct funnel plots if the number of the studies is sufficient and comprises of at least 10 studies.

#### 5.3.11 Data synthesis

A narrative summary together with statistical methods will be used to synthesisthe included studies. Data from the included studies will be grouped according to study characteristics, participants, exercise intervention, outcome measures and study quality. The synthesis will also be presented as a summary table.

Due to expected variation of studies, we will synthesis the effect sizes for each outcome using a random‐effect model with an inverse variance estimation method (Borenstein, Hedges, Higgins, & Rothstein, 2011) to pool outcomes from a sufficiently homogenous set of studies into a meta‐analysis. In order to utilise this model, standard errors are required. Therefore, the odds ratio will be expressed. The analysis will be conducted using RevMan and Comprehensive Meta‐Analysis software (CMA). We will conduct separate metaregression analyses using the CMA software to explore heterogeneity between subgroups based on gender, age, interventions and outcomes.

We will conduct separate meta‐analysis for different study designs and for subcategories of intervention and outcomes. In addition, separate analyses will be conducted for each treatment arm, if a study includes more than one treatment arm compared with control group (if enough studies are found allow to do this). We will conduct separate‐wise comparisons (Higgins & Green, 2011). For dichotomous data, we will sum the sample sizes and events across groups. For continuous data, we will combine sample sizes, means and SDs according to the formula detailed in the *Cochrane Handbook for Systematic Reviews of Interventions* (Higgins & Green, 2011). To account for statistical dependencies, robust variance estimations will be used (Hedges, Tipton, & Johnson, 2010). If the studies report multiple measures of the same construct at different points in time, we will conduct separate meta‐analyses for outcomes measured at a similar time point of follow‐up: short‐term (less than 6 months after intervention), medium‐term (6 months to less than 12 months after intervention) and long term (12 months or more after intervention).

#### 5.3.12 Subgroup analysis and investigation of heterogeneity

Subgroup analysis will be assessed through the comparison of the following: participant demographics (gender, age), type of intervention (group exercises vs. individual exercises) and length of intervention (short duration [week] vs. long duration [years]. Conducting these subgroup analyses will help to establish the generalisability of the effect of exercise programmes by gender, age, type of intervention and length of intervention.

#### 5.3.13 Sensitivity analysis

Sensitivity analysis will be conducted to determine whether the overall results of data analysis are influenced by the removal of:

- unpushlished studies;
- studies with outlier effect size;
- studies with high risk of bias overall;
- studies with missing information.

## CONFLICT OF INTERESTS

The authors declare that there are no conflict of interests.

## AUTHOR CONTRIBUTIONS

Substantial contributions to conception and design: Roongtip Duangkaew, Josette Bettany‐Saltikov, Paul van Schaik, Gok Kandasamy. Study search and selection: Julie Hogg, Roongtip Duangkaew, Josette Bettany‐Saltikov, Paul van Schaik, Gok Kandasamy. Methodological assessment: Roongtip Duangkaew, Josette Betany‐Saltikov, Paul van Schaik, Gok Kandasamy. Acquisition/abstract of data: Roongtip Duangkaew, Josette Bettany‐Saltikov, Pual van Schaik, Gok Kandasamy. Data analysis: Roongtip Duangkaew, Paul van Schaik, Josette Bettany‐Saltikov, Gok Kandasamy. Interpretation of data: Roongtip Duangkaew, Paul van Schaik, Josette Bettany‐Saltikov, Gok Kandasamy. Drafting of the article: Roongtip Duangkaew, Josette Bettany‐Saltikov, Paul van Schiak, Gok Kandasamy. Critical revision for important intellectual content: Roongtip Duangkaew, Josette Bettany‐Saltikov, Paul van Schiak, Gok Kandasamy.

## APPENDIX 1

### MEDLINE search strategy

**Table:**

Search ID	Search terms
S1	"Hyperkyphosis"
S2	"kyphotic posture"
S3	"skeletal alignment"
S4	"skeletal malalignment" OR (MH "Bone Malalignment")
S5	“age‐related hyperkyphosis”
S6	"trunk deformity"
S7	"spinal deformity"
S8	"thoracic hyperkyphosis"
S9	"spinal posture"
S10	(MH "Spinal Curvatures") OR "spinal curvatures"
S11	(MH "Kyphosis") OR "kyphosis"
S12	"Thoracic kyphosis"
S13	S1 OR S2 OR S3 OR S4 OR S5 OR S6 OR S7 OR S8 OR S9 OR S10 OR S11 OR S12
S14	(MH "Exercise") OR "exercise" OR (MH "Exercise Movement Techniques")
S15	(MH "Resistance Training")
S16	(MH "Exercise Therapy")
S17	(MH "Muscle Stretching Exercises") OR "muscle stretching exercises"
S18	"corrective exercise"
S19	"exercise intervention"
S20	"postural correction"
S21	"extension exercises"
S22	(MH "Rehabilitation") OR "rehabilitation"
S23	"strengthening exercises"
S24	"Therapeutic exercises"
S25	"elongation exercises"
S26	"posture correction"
S27	S14 OR S15 OR S16 OR S17 OR S18 OR S19 OR S20 OR S21 OR S22 OR S23 OR S24 OR S25 OR S26
S28	"balance"
S29	"postural sway"
S30	(MH "Postural Balance")
S31	"postural equilibrium"
S32	"centre of pressure"
S33	"centre of pressure"
S34	"body sway"
S35	"limits of stability"
S36	"postural stability"
S37	S28 OR S29 OR S30 OR S31 OR S32 OR S33 OR S34 OR S35 OR S36
S38	(MH "Accidental Falls") OR "falls"
S39	"fall*"
S40	"falling"
S41	"fall risk"
S42	"risk of falls"
S43	"fear of falling"
S44	S38 OR S39 OR S40 OR S41 OR S42 OR S43
S45	"back shape"
S46	(MH "Posture") OR "posture"
S47	"back posture"
S48	"body posture"
S49	"kyphosis angle"
S50	"thoracic kyphosis angle"
S51	"lumbar lordosis angle"
S52	"degree of thoracic kyphosis"
S53	"degree of spinal malalignment"
S54	"spinal angle"
S55	"Cobb angle"
S56	"degree of lumbar lordosis"
S57	"spinal sagittal alignment"
S58	S42 OR S43 OR S44 OR S45 OR S46 OR S47 OR S48 OR S49 OR S50 OR S51 OR S52 OR S53 OR S54
S59	S13 AND S25 AND S34 AND S41 AND S55
